# Mitochondria-associated membranes (MAMs) and inflammation

**DOI:** 10.1038/s41419-017-0027-2

**Published:** 2018-02-28

**Authors:** Sonia Missiroli, Simone Patergnani, Natascia Caroccia, Gaia Pedriali, Mariasole Perrone, Maurizio Previati, Mariusz R. Wieckowski, Carlotta Giorgi

**Affiliations:** 1 Department of Morphology, Surgery and Experimental Medicine, Section of Pathology, Oncology and Experimental Biology, Laboratory for Technologies of Advanced Therapies (LTTA), University of Ferrara, Ferrara, Italy; 2 Department of Morphology, Surgery and Experimental Medicine, Section of Human Anatomy and Histology, Laboratory for Technologies of Advanced Therapies (LTTA), University of Ferrara, Ferrara, Italy; 3 Department of Biochemistry, Nencki Institute of Experimental Biology, Warsaw, Poland

## Abstract

The endoplasmic reticulum (ER) and mitochondria are tightly associated with very dynamic platforms termed mitochondria-associated membranes (MAMs). MAMs provide an excellent scaffold for crosstalk between the ER and mitochondria and play a pivotal role in different signaling pathways that allow rapid exchange of biological molecules to maintain cellular health. However, dysfunctions in the ER–mitochondria architecture are associated with pathological conditions and human diseases. Inflammation has emerged as one of the various pathways that MAMs control. Inflammasome components and other inflammatory factors promote the release of pro-inflammatory cytokines that sustain pathological conditions. In this review, we summarize the critical role of MAMs in initiating inflammation in the cellular defense against pathogenic infections and the association of MAMs with inflammation-mediated diseases.

## Facts

- MAMs are a molecular platform essential for NLRP3 inflammasome formation.
- The adaptor MAVS is required for NLRP3 mitochondrial localization and promotes its activation.
- MAMs are directly involved in DAMP generation and in the cellular antiviral response.
- Several MAM-resident proteins such as α-synuclein and presenilins play a crucial role in the pathogenesis of Parkinson’s disease and Alzheimer’s disease, respectively.

## Open questions

- How can such a small subcellular organelle have such an important function in NLRP3 inflammasome formation?
- Could MAMs be considered a potential target for pathogenic bacteria to disrupt inflammasome activation and signaling during infection?
- How could MAMs be developed to target or treat diseased conditions?
- Is it feasible to assume that the integrity and functions of MAMs are relevant for therapeutic intervention?

## Introduction

Studies over the past few decades have demonstrated that the endoplasmic reticulum (ER) and mitochondria are physically connected to form junctions termed mitochondria-associated membranes (MAMs).

The MAMs fraction was first identified by J.E. Vance^1^ in 1990, who described the isolation of a unique membrane “fraction X” from rat liver enriched in a protein implicated in lipid synthesis and trafficking (for a detailed review, see ref. 2).

Several biochemical techniques since have been described to isolate the MAM fraction, confirming that MAMs are composed of membrane fragments from both the ER and the outer mitochondrial membrane (OMM)^3^.

In the past few years, different proteomics studies have identified the molecular components of the MAM fraction, demonstrating both from human fibroblast^4^ and from mouse brain^5^ that >1000 “MAM proteins” reside in this fraction. Recently, Sala-Vila et al. performed in-depth mass spectrometric analysis of the proteins composing MAM-enriched fractions and identified 1052 proteins including caveolin-1 (CAV1), which is an integral component of hepatic MAMs that determines the relative cholesterol content of these ER subdomains^6^.

MAMs provide a platform that is fundamental for several cellular functions, such as calcium (Ca^2+^) homeostasis, autophagy, lipid metabolism, apoptosis, and tumor growth^7,8^, that allow rapid exchange of biological molecules to maintain cellular health. Therefore, the accurate connections and crosstalk between the ER and mitochondria are events that coordinate important functions of the two organelles and thus determine key aspects of cell fate. In fact, alterations in the composition of MAMs and the abnormal induction of this ER–mitochondria association lead to different pathological conditions^9^.

For these reasons, several regulatory proteins, oncogenes, and tumor suppressors reside at MAMs in order to maintain normal cellular function and thereby preserve the intracellular equilibrium^10,11^.

Considering that the ER–mitochondria interface is involved in several molecular pathways, it is not surprising that MAMs play an emerging role also in inflammatory signaling pathways. In fact, MAMs provide a critical site for inflammasome formation, are attractive targets for pathogenic bacteria and are relevant for the antiviral response (see the next paragraphs).

Additionally, increasing evidence strongly implicates the involvement of MAMs in the initiation or progression of diseases associated with high inflammation, such as metabolic disorders and neurodegenerative diseases^12^.

Finally, recent developments of promising therapeutics that target inflammasome activities at MAMs in inflammatory diseases have been reported. In this review, we discuss how MAMs represent a primary platform for initiating inflammatory mechanisms and how these networks might be manipulated to provide novel therapies for inflammatory diseases.

## MAMs as a critical site for inflammasome formation

A link between the ER–mitochondria interface and inflammation was first recognized a few years ago with the observation that reactive oxygen species (ROS) promote the activation of NOD-like receptor protein 3 (NLRP3) inflammasomes^13^.

The inflammasome is a multiprotein complex composed of a sensor protein, an adaptor protein called apoptosis-associated speck-like protein, containing a caspase-recruitment domain (ASC) and pro-caspase 1, a cysteine protease. There are four subfamilies of inflammasomes depending on the sensor molecule: NLRP3, NLRP1, NLRC4 (NLR family, CARD domain containing 4), and AIM2 (absent in melanoma 2)^14^.

These sensor molecules can sense diverse stimulators ranging from microbial products (pathogen-associated molecular patterns, PAMPs) to host-derived damage signals (damage-associated molecular patterns, DAMPs) or any other insults that may occur within the cytosol. Once assembled, pro-caspase 1 is autoprocessed by proximity to active caspase 1, which induces the maturation of pro-interleukin-1β (IL-1β) or pro-IL-18 to their activated form. Among the inflammasomes, NLRP3 is the most studied and characterized due to its implication in the pathogenesis of different human diseases^15^. To date, the NLRP3 complex is the only inflammasome complex to be described as associated with MAMs^13^.

Despite receiving much attention and investigation^16,17^, the activation of the NLRP3 inflammasome remains an enigmatic mechanism. So far, a two-step process has been proposed for the activation of NLRP3: (i) a priming step triggered by the interaction of Toll-like endosomal receptors (TLRs) with their ligands, leading to the transcription of NLRP3 and pro-IL-1β by nuclear factor kB (NF-κB), and (ii) an activation step promoted by several stimulators that initiates complex assembly in a manner dependent on K^+^ efflux through P2×7 activation^18^, lysosomal rupture, or mitochondrial ROS^19^. Moreover, different models have been proposed to explain the role of Ca^2+^-signaling in NLRP3 activation (for a detailed review, see ref. 20). Lee et al. proposed a critical role for calcium-sensing receptor (CASR) in activating the NLRP3 inflammasome that is mediated by increased intracellular Ca^2+^ and decreased cellular cyclic AMP (cAMP)^21^. An alternative model proposed that sustained Ca^2+^ influx (via ER Ca^2+^ release channels) triggers mitochondrial Ca^2+^ overload and associated mitochondrial destabilization^22^ and is characterized by high levels of ROS production and the induction of mitochondrial permeability transition pore (mPTP)^23^. Supporting this model, Misawa et al. observed that during inflammasome formation microtubules drive the perinuclear migration of mitochondria that results in subsequent apposition of ASC on mitochondria to NLRP3 on the ER^24^.

**Fig. 1:**
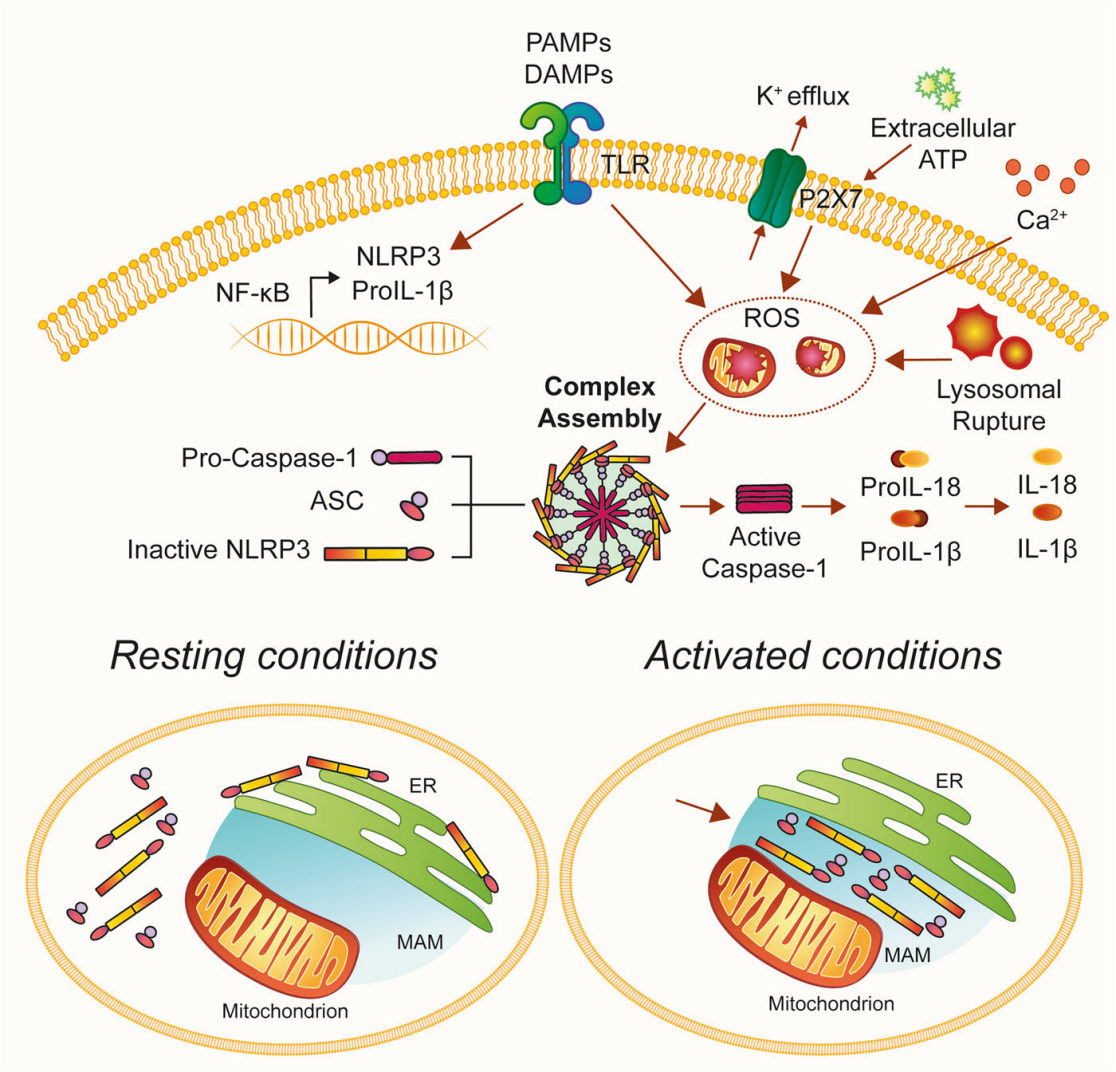
Mitochondria-associated ER membranes are important sites for NLRP3 inflammasome activation Under non-stimulatory conditions, most NLRP3 protein localizes to cytoplasmic granular structures. Stimulation with extracellular ATP and particulate/crystalline activators, which lead to lysosomal rupture, DAMPs, and PAMPs, triggers the generation of ROS that activate NLRP3. Once activated, NLRP3 recruits an adaptor protein called apoptosis-associated speck-like protein containing a CARD (ASC) and redistributes to the MAM fraction. Thus, upon pro-inflammatory stimuli, NLRP3 oligomerizes and exposes its effector domain to interact with ASC, which in turn recruits pro-caspase-1. Finally, activated caspase-1 cleaves pro-IL-1β to generate mature IL-1β. ASC apoptosis-associated speck-like protein containing a CARD, IL-1β interleukin-1 beta, NLRP3 NOD-like receptor protein 3

NLRP3 is expressed in most tissues but predominantly in macrophages. In the inactive state, NLRP3 localizes to the ER membrane and cytosol, but when activated both NLRP3 and its adaptor ASC relocate to the MAM fraction (Fig. 1) where they detect increased ROS production from damaged mitochondria^13^. In particular, respiratory chain inhibitors (like complex I inhibitor rotenone) activate the inflammasome implicating a loss of the ΔΨm and ROS accumulation. Therefore, by virtue of its ER–mitochondria localization upon activation, the NLRP3 inflammasome is strategically located to sense signals emanating from mitochondria.

Consistent with these observations, defective mitophagy has been shown to enhance inflammasome activation. In fact, inhibition of mitophagy/autophagy by 3-methyladenine (3MA) treatment or silencing the autophagy regulator Beclin 1 and autophagy protein 5 (ATG5) in macrophages resulted in increased NLRP3 activation and IL-1β release upon stimulation with monosodium urate (MSU) crystals and nigericin due to the accumulation of damaged mitochondria and increased ROS generation^25^. Moreover, treatment with the antioxidant 4-amino-2,4-pyrrolidinedicarboxylic acid (APDC) blocked NLRP3 inflammasome activation and IL-1β secretion^13^.

Interestingly, a study by the group of M. Karin demonstrated that NF-κB could exert its anti-inflammatory activity and restrain NLRP3 inflammasome activation, by inducing delayed accumulation of the autophagy receptor p62/SQSTM1^26^. p62, induced on NF-κB activation, suppresses NLRP3-inflammasome-dependent IL-1β production.

Therefore, the “NF-κB–p62/SQSTM1–mitophagy” pathway provides a crucial regulatory loop through which NF-κB orchestrates NLRP3 inflammasome activation and cytokine release thereby focusing on a reparative inflammatory response and preventing excessive collateral damage^26^.

However, the exact role of autophagy on the secretion of IL-1β is still a matter of intense study and debate^27^. Indeed increasing data from the literature support the idea that induction of autophagy can directly promote IL-1β secretion after inflammasome activation by incorporating it into the autophagosomal carrier^28^. Conversely, autophagy inhibition induces interleukin-1β accumulation inside the cells blocking its release^28^.

Therefore, a better understanding of the NLRP3 inflammasome is required for the development of effective therapeutic treatments for NLRP3-related inflammatory diseases.

Additionally, mitochondrial antiviral-signaling protein (MAVS), best known for its role in the innate immune system (see next section for further details), has been suggested to recruit NLRP3 to mitochondria in response to viral infection^29^. This study by Subramanian N. demonstrated that MAVS is required for optimal NLRP3 inflammasome activity and identified the N-terminal amino acid sequence in NLRP3 that is fundamental for its association with MAVS at the mitochondria^29^.

Another NLRP3-binding partner thioredoxin-interacting protein (TXNIP) redistributes to MAMs/mitochondria in response to oxidative stress^30^ or NLRP3 inflammasome activation^31^. In resting cells, TXNIP interacts with thioredoxin (TRX, a cellular antioxidant protein) and is therefore unavailable for interaction with NLRP3. Inflammasome activators, such as uric acid crystals, induce the dissociation of TXNIP from oxidized TRX in a ROS-sensitive manner and allow it to bind NLRP3 and translocate to MAMs/mitochondria. This raises the possibility that TXNIP is involved in IL-1β production through NLRP3 under ER stress conditions^31^.

Together with ROS, mitochondrial DNA (mtDNA) is one of the mitochondrial damage signals that interacts directly with NLRP3 and AIM2, and oxidized mtDNA (ox-mtDNA) interacts specifically with NLRP3 in cells with ATP and nigericin treatment^32^. Moreover, IL-1β release is drastically reduced in AIM2 KO macrophages in response to mtDNA. Importantly, IL-1β release is higher after ox-mtDNA stimulation than mtDNA, and AIM2 does not seem to be involved in ox-mtDNA–mediated inflammasome activation^32^.

Although these numerous studies support the idea that mitochondrial dysfunctions and ROS are closely associated with inflammasome assembly and activation, there is a lack of understanding how these factors trigger NLRP3 activation.

Recently, multiple works have focused on studying the possible pathway and activation mechanisms of the other inflammasomes. For instance, D’Osualdo et al. showed that expression of NLRP1, a core inflammasome component, is specifically upregulated during severe ER stress conditions in human cell lines^33^, which suggests a plausible involvement of MAMs.

All these data highlight the importance of the MAM interface as a platform for inflammasome formation and require additional studies to identify the mechanism that induces inflammasome activation at this critical cellular site.

## Antiviral response at the MAM interface

In addition to their established role, MAMs can exert a special role in the immune-viral response^34,35^.

The innate immune response is regulated by several germline-encoded receptors, called pattern recognition receptors (PRRs), which are able to detect PAMPs and DAMPs. In particular, TLRs and cytosolic sensors of the RIG-I-like receptor family (RLRs) can also sense the cell’s double-stranded DNA and the DNA derived from viral infection, respectively, when present in the cytosol, or single-stranded viral RNA^34,36^.

**Fig. 2:**
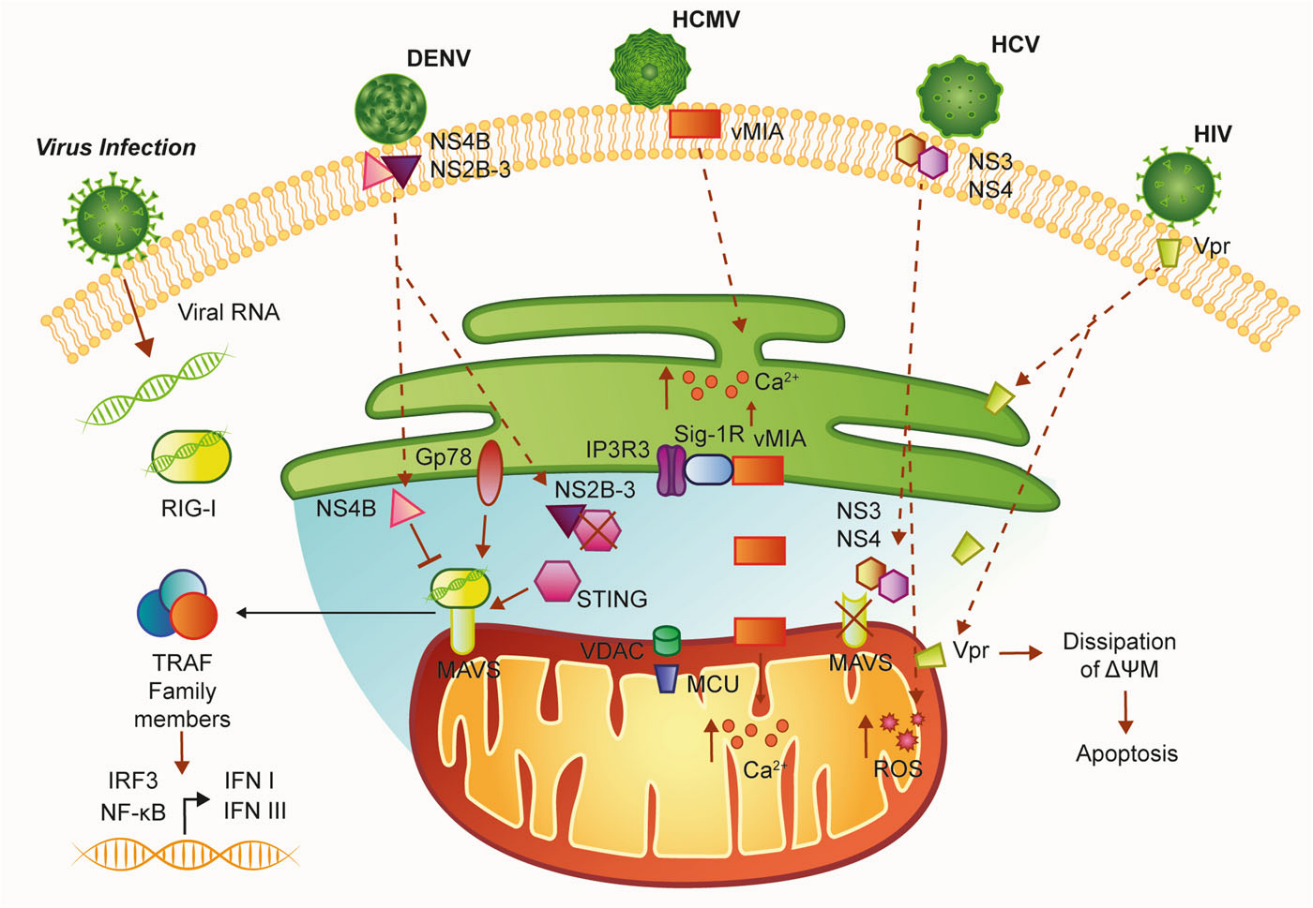
Schematic representation of MAMs in the antiviral response MAVS is located on the outer mitochondrial membrane (OMM) and mediates antiviral signaling by inducing the recruitment of several members of the TRAF family. Moreover, MAVS interacts with the helicases RIG-1 or MDA5 (melanoma differentiation-associated gene 5). Upon virus infection, MAVS and RIG-I create a complex with STING that increases the interferon response. Gp78 can be considered another MAVS interactor that regulates MAVS-mediated antiviral signaling. The UL37 protein from HCMV traffics into the MAMs during permissive infection and induces alteration of the Ca^2+^ signaling. Infection with HIV-1 directly targets MAMs leading to mitochondrial polarization and apoptosis. Infection with DENV results in the compromised integrity of MAMs and reduced RIG-1-dependent IFN response. DENV dengue virus, ER endoplasmic reticulum, HCMV human cytomegalovirus of the large family of DNA viruses Herpesviridae, HCV hepatitis C virus, HIV-1 immunodeficiency virus 1, I IFN interferon I, IRF3 interferon regulatory factor 3, MAVS mitochondrial antiviral-signaling protein, MCU mitochondrial Ca^2+^ uniporter, NF-kB nuclear factor-κB, NS3 nonstructural protein 3, NS4 nonstructural protein 4, RIG-I retinoic acid-inducible gene-I protein, ROS reactive oxygen species, Sig1-R sigma-1 receptor, STING Stimulator of interferon genes, Vpr viral protein R

The RIG-I-like receptor family includes the retinoic acid-inducible gene-I (RIG-I) protein, the melanoma differentiation-associated gene 5 (MDA5) protein, and the RIG-I-like receptor LGP2, and this receptor family triggers damage signaling by sensing viral RNA. These proteins share several homologous domains, including the presence of caspase activation and recruitment domains (CARDs) and a DEAD box helicase/ATPase domain^37^. In particular, the CARD allows RIG-I and MDA5 to interact with MAVS. MAVS is localized at the mitochondrial membrane through its C-terminal transmembrane domain. The MAVS mitochondrial localization is required to trigger the downstream antiviral signaling pathways^38^. In fact, MAVS activation induces the recruitment of several members of the TRAF family, followed by phosphorylation and nuclear translocation of interferon regulatory factor 3 (IRF3), as well as activation of NF-κB; this induces transcription of interferon I (I IFN) and III genes and other inflammatory cytokines^39^ (Fig. 2).

Stimulator of interferon genes (STING) has a central role in controlling immune responses to cytoplasmic DNA. STING activation depends on the binding of a type of cyclic dinucleotide (CDN) termed cGAMP (cyclic GMP–AMP) by GMP-AMP synthase (cGAS)^40^. This leads to the phosphorylation of IRF3 and NF-κB and the subsequent induction of cytokines and proteins, such as the type I IFN, that exerts its antipathogenic activities^41,42^. Although these pathways, in principle, detect different signals, crosstalk between these distinct signaling pathways has been reported to be important. In several studies, STING appeared to interact with RIG-I and MAVS in a complex that was stabilized upon virus infection^43^. STING can bind MAVS at MAMs thus increasing the interferon response to viral infection^43,44^, whereas genetic ablation of STING inhibited the activation of the IRF/IFN pathway in the presence of the ssRNA genome of the Japanese encephalitis virus^45^. The physical interaction between STING and MAVS for microbial DNA and RNA recognition is of particular interest for several reasons: (i) converges the two signaling pathways at the mitochondrial level, where MAVS is anchored; (ii) incorporates MAMs into antiviral signaling, and (iii) orients viral strategies to dampen or prevent the activation of the IFN response and also toward MAM dysregulation. In fact, the alteration of the contacts between the ER and mitochondria and destruction of the MAMs/mitochondria-associated MAVS is a common strategy for many viruses.

Lastly, Gp78, an E3 ubiquitin ligase which is active in the ER-associated degradation (ERAD) pathway, has been considered a novel regulator of RLR signaling that localize to the ER–mitochondria interface^46^. Gp78 physically interacts with MAVS and regulates MAVS-mediated antiviral signaling through two possible mechanisms. The first requires its E3 ubiquitin ligase and ERAD activity to directly degrade MAVS, while the second occurs independent of these activities, requires the Gp78 RING domain, and occurs via a direct association between this region and MAVS^46^.

Taken together, these data suggest that other MAM-localized components might also serve to specifically target MAVS as a means to regulate inflammatory signaling within the cell. Defining the detailed components of the MAVS regulome specifically within the MAMs will certainly provide exciting new insights into the regulation of antiviral signaling.

Given the important role of the MAMs in the antiviral response, it is not surprising that numerous viral proteins target this structure (Fig. 2). One well-characterized example is vMIA (also named pUL37×1), which is an immediate-early protein synthesized by HCMV, the human cytomegalovirus of the large family of DNA viruses Herpesviridae. This protein exerts a potent antiapoptotic function in infected cells^47^. vMIA is initially synthesized in the ER and localizes on the ER membrane. However, the presence of a moderately hydrophobic leader peptide retargets all isoforms of vMIA to the MAMs and OMM^4,48,49^. This association of vMIA to MAMs allows the virus to exploit cellular level cytotoxic effects, such as the prevention of the antiviral response. Specifically, the additional presence of a consensus cholesterol-binding domain allows vMIA to associate with detergent-resistant membranes at the MAMs, without affecting the OMM localization^49^. The cholesterol-dependent association of vMIA with MAMs is responsible for at least part of the toxic action of the virus because it allows vMIA to interact with the sigma-1 receptor (Sig-1R); this contributes to the regulation of inositol 1,4,5-triphosphate receptor (IP3R) at the ER/MAM interface, thus influencing their Ca^2+^ transfer and degradation^50–52^. In addition to the regulation of IP3Rs, vMIA affects Ca^2+^ transfer increasing the expression of mitochondrial Ca^2+^ uniporter (MCU), MICU1, ER pump SERCA, and VDAC^4,53,54^, contributing to overload of Ca^2+^ entry in the mitochondria and inducing a condition of programmed cell death.

To prevent the cellular antiviral response, vMIA deeply affects mitochondrial morphology by both increasing mitochondrial biogenesis^55^ and by causing mitochondrial fragmentation in transfected cells and during viral infection^56–60^. Consequently, this results in the reduction of the number of MAMs and of the interactions between MAVS and STING. These alterations thereby reduce the downstream signaling against HCMV infection^60^.

Furthermore, HMCV re-localizes various cellular proteins, including calreticulin, calnexin, PACS-2, and glycolytic enzymes, to the MAMs platform. The whole effect is to induce a metabolic reprogramming that reduces pyruvate utilization at mitochondria level but increase the metabolic intermediates for biosynthetic reactions^61,62^.

MAVS activity is a not only a target of HCMV but also of hepatitis C virus (HCV), a small enveloped single-stranded, positive-sense RNA virus. Upon interaction with HCV viral RNA, RIG-1 tetramerizes and recruits MAVS, which in turn elicits an IFN-mediated antiviral response. To avoid this, the multifunction HCV serine proteases residing in nonstructural protein 3 (NS3) and 4 (NS4) can cleave MAVS. NS3/4A can associate to intracellular membranes through their membrane-targeting domains within NS4A and the amphipathic α helix of NS3 and thus target MAVS specifically localized at MAMs^39,63^. So, an HCV viral strategy, based on protease inactivation of MAVS, leads to the suppression of type I and III IFN production^39^. Other HCV proteins have been found to localize to MAMs^64^, where they could be responsible for the documented elevation of mitochondrial ROS by manipulating Ca^2+^ entry, in particular at the level of MCU^65–67^.

Another example of a virus that directly targets MAMs is the human immunodeficiency virus 1 (HIV-1) lentivirus, a single-stranded positive-sense RNA virus that can have a strong cytotoxic effect on host cells, in particular on human primary CD4+T cells^68^. This event occurs after insertion of viral protein R (Vpr) into the membranes of the ER, OMM, and MAMs. This insertion can dissipate the mitochondrial transmembrane potential and lead to apoptosis. Membrane insertion of Vpr is obtained through its C-terminal transmembrane hydrophobic segment, which shares homology with proteins of Myxoma M11L, vaccinia F1L, Epstein-Barr BHRF-1, and HCVs, and contains mitochondrial targeting sequence^69–71^. In particular, it has been found that Vpr transport occurs from the ER to OMM through MAMs and suggests the presence of dynamin-related protein 1 (Drp1), Mitofusin 2 (Mfn2), and ATPase family, AAA domain containing 3A^72^. As a consequence of Vpr interaction, DRP-1 and Mfn2 are downregulated via the VprBP-DDB1-CUL4A ubiquitin ligase complex, and mitochondria showed fragmentation and disruption of OMM^73^.

Additionally, dengue virus (DENV) carries out a strategy similar to HCMV and HCV. The mosquito genus Aedes is the host for this RNA virus of the Flaviviridae family that infects 10 million cases per year worldwide. DENV promotes infection by altering mitochondrial morphology and weakening the IFN response at the MAMs. DENV replicates at ER-derived cytoplasmic structures, such as the convoluted membranes (CM)^74,75^. The DENV nonstructural protein NS4B induces elongation of mitochondria, which is associated with the downregulation of Drp1. Elongated mitochondria physically contact CMs and exhibit compromised MAMs integrity and reduced RIG-1-dependent IFN response^74^. In particular, NS4B co-localizes with MAVS in the MAMs by interacting with the N-terminal CARD-like domain and the C-terminal transmembrane domain of MAVS. This association prevents the binding of MAVS to RIG-I and results in the suppression of RIG-I-induced IRF3 activation and, consequently, the abrogation of IFN production^74^. Similar to HCV, alternative strategies of DENV include the proteolytic inactivation of MAM-resident proteins. DENV NS2B-3 protease, which is highly enriched in CMs, proteolytically inactivates the MAM-resident signaling adaptor STING^76^ and prevents RIG-I translocation to mitochondria by targeting the adaptor protein 14-3-3ε using a highly conserved phosphomimetic motif^77^. Taken together, these studies highlight the importance of MAMs in the antiviral response.

## MAMs are attractive targets for pathogenic bacteria

**Fig. 3:**
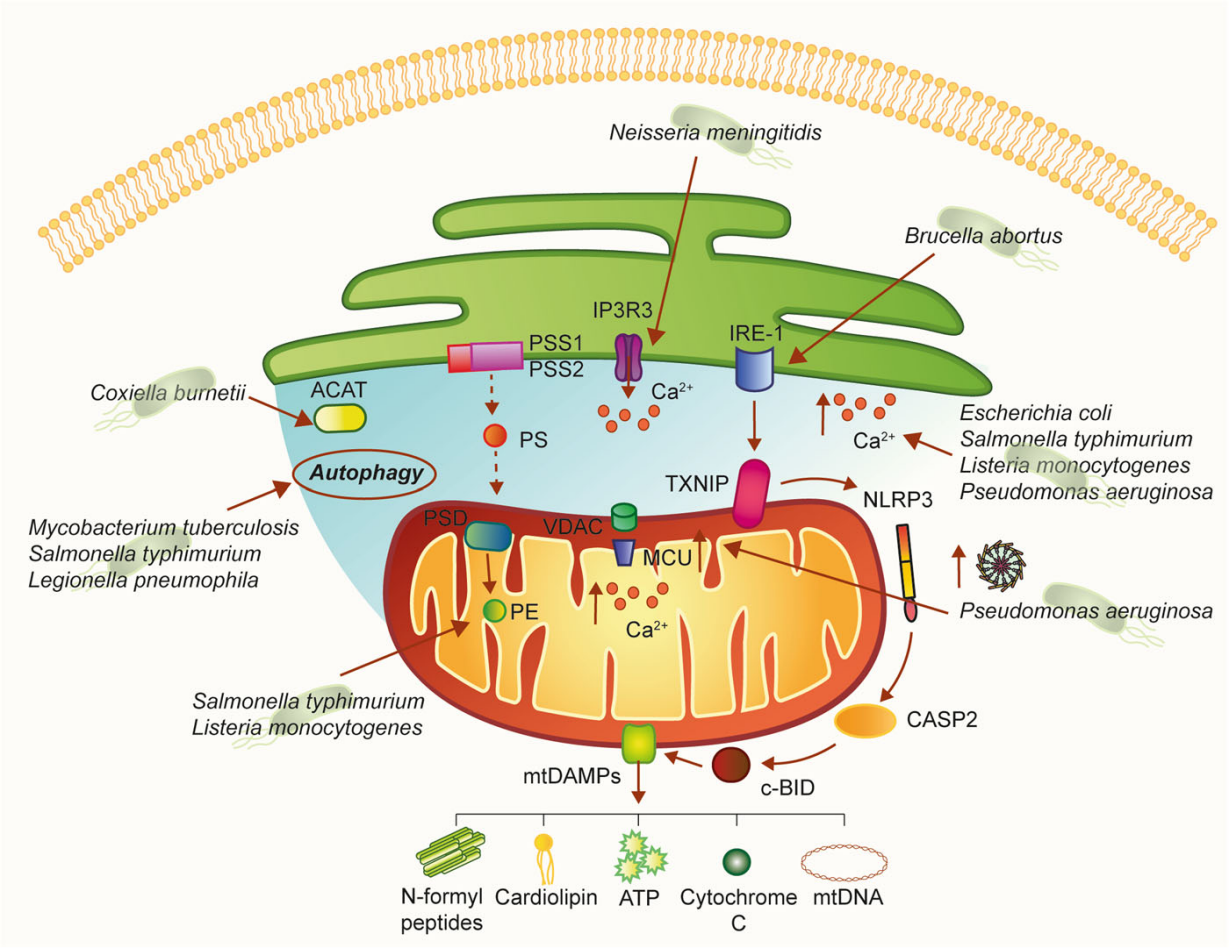
A hypothetical model of how bacterial pathogens could affect MAM-mediated cellular processes MAMs are a molecular platform involved in several cellular processes. As such, different pathogenic bacteria could alter these signaling pathways. Interestingly, once released into the extracellular space, mitochondrial DAMPs of bacterial origin, such as cardiolipin and NFPs, can stimulate the innate and adaptive immune responses. ACAT enzyme acyl-CoA:cholesterol acyltransferase, CASP2 caspase 2, Ca^2+^ calcium, IP3R3 inositol 1,4,5-triphosphate receptor type 3, MCU mitochondrial Ca^2+^ uniporter, NFPs N-formyl peptides, NLRP3 NOD-like receptor protein 3, PE phosphatidylethanolamine, PS phosphatidylserine, PSD phosphatidylserine decarboxylase, PSS1 phosphatidylserine synthase-1, PSS2 phosphatidylserine synthase-2, Sig1-R sigma-1 receptor, TXNIP thioredoxin-interacting protein

Among the numerous processes coordinated by MAMs, inflammatory signaling pathways associated with MAMs play critical roles in the cellular defense against pathogenic infections (Fig. 3). MAMs provide an excellent platform for the coordination of lipid synthesis and trafficking, mitochondrial morphology, and autophagosome formation^78^. Additionally, all these important cellular processes are modulated by bacterial pathogens to suppress host functions and promote infections; in fact, pathogenic bacteria frequently target the ER and mitochondria to carry out host functions. Among these bacterial pathogens, intracellular bacteria, such as *Salmonella typhimurium*, *Chlamydia trachomatis*, *Mycobacterium tuberculosis*, *Listeria monocytogenes*, *Legionella pneumophila*, or *Yersinia pestis*, have the ability to grow and replicate inside host cells. MAMs could be targeted by pathogenic bacteria to subvert key host cellular processes^79^. However, why are MAMs such attractive targets for pathogenic bacteria?

MAMs coordinate the synthesis and transformation of phosphatidylserine (PS) into phosphatidylethanolamine (PE), which is one of the most abundant phospholipids in bacterial cell membranes. The two enzymes phosphatidylserine synthase-1 (PSS1) and 2 (PSS2) are highly enriched in MAMs^80^ and are responsible for PS synthesis. After being transported to the mitochondria, PS is decarboxylated into PE by the enzyme phosphatidylserine decarboxylase (PSD) located in the inner mitochondrial membrane (IMM).

PE can be readily broken down into glycerol and ethanolamine by phosphodiesterases, and bacteria take advantage of ethanolamine as a nutrient source^81^. Interestingly, S. *typhimurium* in the lumen of the inflamed intestine use PE-derived ethanolamine as a carbon source to produce a respiratory electron acceptor (tetrathionate). This supports anaerobic growth with ethanolamine, which is released from host tissue but is not utilizable by competing bacteria^82^. Interestingly, the contribution of ethanolamine utilization to *L. monocytogenes* pathogenesis, in an intravenous mouse infection model, suggests that ethanolamine utilization is important outside of the intestine and possibly in the intracellular environment^83^.

Phospholipid synthesis is one of the major functions of MAMs. Given the enzyme acyl-CoA:cholesterol acyltransferase (ACAT) is highly enriched at MAMs, it has been suggested that MAMs might also serve as a site for cholesterol synthesis and neutral lipid synthesis^84^. *Coxiella burnetii* require lipids for both normal bacterial functions as well as formation of the acidic, phagolysosomal-like parasitophorous vacuole (PV) surrounding the bacteria. *C. burnetii* does not have the capability to generate cholesterol and thus utilizes host cell lipids for membrane biogenesis and possibly energy. This suggests that sterols are actively diverted from the host cell^85^.

Since the central role of MAMs in Ca^2+^ homeostasis, which in turn is key for the initiation of apoptosis^86^, and considering that bacterial pathogens can modulate Ca^2+^ fluxes as a strategy for pathogenesis (for a detailed review, see ref.^87^), it is plausible to think that MAMs might be involved in the subversion of apoptosis and Ca^2+^ signaling during bacterial infections. Just to mention a few examples, bacterial toxins from pathogens such as *Escherichia coli* or *L.* *monocytogenes*, as well as flagellins of *S. typhimurium* and *Pseudomonas aeruginosa*, can induce an increase in free cytosolic Ca^2+^ in host cells, required for the toxin-mediated effects. Rimessi et al. demonstrated that *P. aeruginosa* affects Ca^2+^ signaling and mitochondrial function, in which flagellin is the inducer and MCU is a signal-integrating organelle member for NLRP3 activation and IL-1β and IL-18 processing^88^. Interestingly, *Neisseria meningitidis* mediates Ca^2+^ release by activating the IP3 receptors on the surface of the ER, promoting adherence and invasion into host endothelial cells^89^.

Notably, MAMs are the sites where autophagosome formation occurs. This is due to key factors of the initiating machinery of autophagy, such as ATG14 and ATG5, redistributing to MAMs upon autophagy induction^90^. Certain intracellular pathogens such as *M. tuberculosis* and *S. typhimurium* target and inhibit the autophagy response of the host cells during infection^79^; additionally, *L. pneumophila* restrains autophagy by the secretion of bacterial effectors^91^.

Taken together, these and other data demonstrated that bacterial pathogens can modulate some cellular processes in which MAMs are involved; however, whether pathogenic bacteria might target MAMs to disrupt these pathways has not been demonstrated yet.

Another pivotal role of MAMs is in the generation of DAMPs within host cells in response to cellular damage triggered by cellular stress. DAMPs derived from mitochondria, such as ATP, cardiolipin, cytochrome *C*, N-formyl peptides (NFPs), succinate, and others, play a central position as modulators of inflammation during different pathologies and have a central role in the activation of inflammation via NLRP3^92^. Moreover, ROS derived from mitochondria can interact with and directly modify the function of DAMPs that, in turn, can regulate immune responses and contribute to the development of inflammatory diseases^92^.

An elegant study by Bronner et al. revealed that microbial infection with the *Brucella abortus* strain RB51 induced NLRP3 inflammasome activation leading to the induction of mitochondrial DAMPs^93^. This model of infection induced the activation of the ER stress marker IRE-1 that consequently promotes TXNIP1 translocation to mitochondria, which in turn promotes mitochondrial ROS production. This event leads to NLRP3-mediated crosstalk between ER and mitochondria, resulting in the release of mitochondrial contents through activation of the caspase-2–Bid signaling axis^93^.

In particular, the expression of TXNIP induced by ER stress is under the control of the IRE1α and PERK–eIF2α pathways of the UPR^94^. In different studies, it has been proposed that ER stress activates the NLRP3 inflammasome in a K^+^ efflux- and ROS-dependent manner that may also affect the mitochondria, suggesting the critical role played by the MAM as a site for signals exchange between the two organelles.

As previously described, TXNIP1 and IRE1a localize at MAMs^31,95^.

Under ER stress or after exposure to high concentrations of ROS, IRE1 is stabilized at the MAMs by Sig-1R^95^, and this enhanced cellular survival by prolonging the activation of the IRE1–XBP1 signaling pathway. Infection was often associated with ER stress, and animals deficient in components of the IRE1 signaling pathway were more susceptible to bacterial infection than controls^96–98^.

These findings indicate the direct involvement of MAMs in the generation of DAMPs and give rise to further speculations. Exploration of this platform during bacterial infection ensures promising and stimulating new research opportunities.

### Role of MAMs in inflammatory diseases

Considering what has been previously outlined, it is not surprising that members of the inflammatory response are strongly implicated in the initiation or progression of different human pathologies. In particular, inflammatory response proteins have been most commonly implicated in neurodegenerative diseases, which may be attributed to different causes. For example, continuous release of IL-1β negatively modulates the integrity of the brain–blood barrier, which results in the infiltration of immune cells into the central nervous system^99^. The same cytokine amplifies the generation of other pro-inflammatory factors by stimulating the activation of microglia and astrocytes^100^. Moreover, it has been demonstrated that overexpression of IL-1β mediates neuronal injury and cell death throughout glutamate excitotoxicity^101^. An important link between inflammation and neurodegeneration could be found in the fact that the maintenance of the integrity of ER compartment is critical for the conservation of an appropriate MAMs network and functionality. Notably, misfolded protein aggregates and excessive accumulation of metabolites are critical determinants for the activation of ER-stress and NLRP3 inflammasome and, at the same time, are specific hallmarks of initiation and progress of neurodegeneration^15^.

**Fig. 4:**
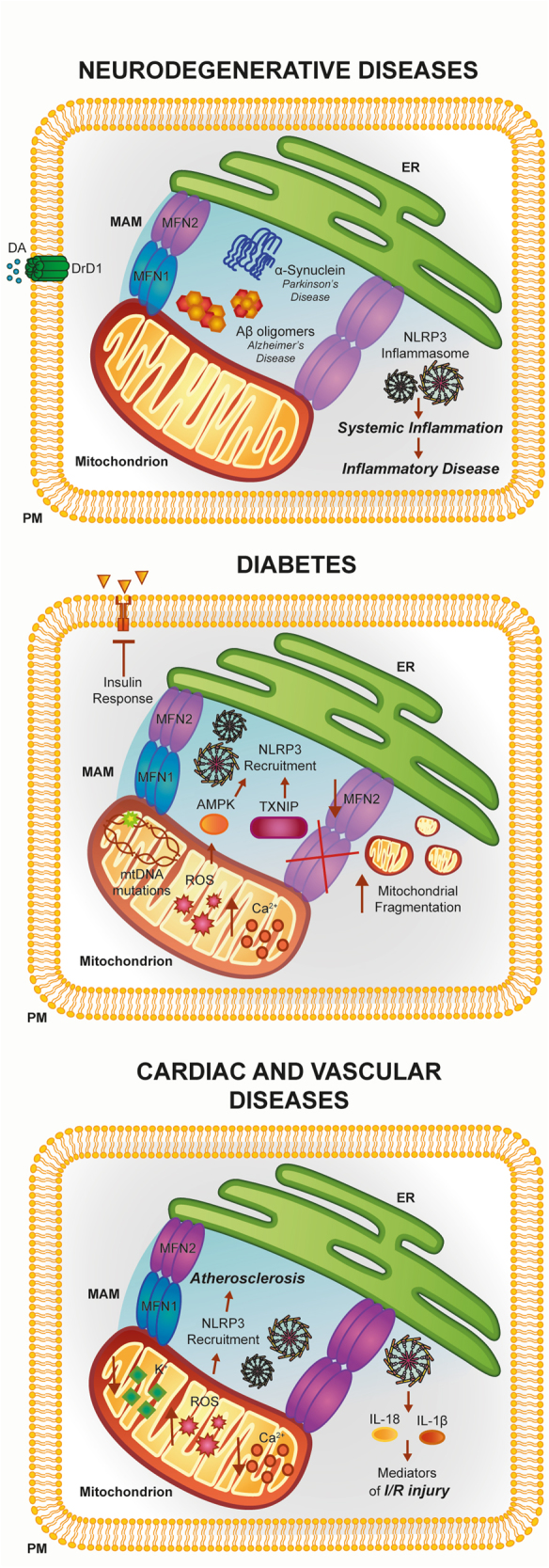
Involvement of the ER–mitochondria interface in the pathogenesis of neuronal disorders, diabetes, and cardiac and vascular diseases The activities of several MAM proteins linked to inflammation have been found to be disrupted during the pathogenesis of a number of human diseases. See text for further details

A classic example may be found in Alzheimer’s disease (AD) that is characterized by aberrant accumulation of amyloid-β plaques in the cerebrum. Indeed, amyloid-β is a potent trigger for ER stress and ROS production^102^. In addition, it has been recently demonstrated that amyloid-β precursor and its catabolites also localize to the MAMs compartment, where they interact with MAM-resident proteins and modulate ER functions^103^. Considering all these aspects, it is easy to speculate that amyloid-β, ROS, and MAMs may be a platform sufficient to trigger the inflammatory processes (Fig. 4).

Intriguingly, amyloid-β was the first peptide associated with neurodegeneration to be shown to activate NLRP3 and promote the production and release of pro-inflammatory cytokines^104^.

These processes appear to be driven by the lysosomal protease cathepsin B. Indeed, during the phagocytosis of amyloid-β by microglial cells, lysosomes that have sequestered this protein lose their function and integrity, resulting the release of lysosomal components. Among these, cathepsin B was found to be necessary to mediate IL-1β production.

This discovery also displays important therapeutic aspects. Remarkably, it has been observed that, throughout inhibition of cathepsin B, it is possible to reduce the numbers of amyloid plaques and improve the memory in the AD mouse model^104^.

Other findings highlight the intimate relationship between amyloid-β and NLRP3. Recently, it has been observed that transgenic mice *Nlrp3*^*−/−*^ or *Casp1*^*−*^^*/−*^ with mutations associated with familial AD have reduced accumulation of amyloid-β, loss of spatial memory, and present enhanced tissue remodeling^105^. Accordingly, excessive levels of caspase-1 were found in the brains of AD patients.

Parkinson’s disease (PD) is a neurodegenerative disease characterized by an excessive death of neurons in the substantia nigra pars compacta (SNpc) caused by α-synuclein (αSyn) aggregates. The majority of αSyn resides in the cytoplasm. However, different investigations show the presence of this protein in the mitochondrial compartment in the striatum, SNpc, and cortex of PD brains, impairing the function of mitochondrial electron chain complexes^106,107^. Most recently, it has been shown that αSyn also localizes at the MAMs^108^. Additionally, in cells and animal models of PD with pathogenic mutations of α-syn, MAM functions are downregulated and, most importantly, that inflammasome is activated^108,109^.

Indeed, studies show that αSyn aggregates are sufficient to provoke IL-1β production by activating microglia and astrocytes. In addition, it has been demonstrated that there is an important difference between the fibrillar and monomeric form of this protein. Meanwhile, the monomeric αSyn only induces the expression of pro-IL-1β. The fibrillary form is able to provoke caspase-1 activation and maturation of IL-1β and thus fully activates the inflammasome. Interestingly, these findings have also been found *in vivo*. Indeed, transgenic mice lacking NLRP3 are resistant to developing PD following treatment with the neurotoxin 1-methyl-4-phenyl-1,2,3,6-tetrahydropyridine (MPTP). Despite this, the endogenous regulatory mechanisms of inflammasome activation in PD are still unclear. A role for ROS and cathepsin-B has also been suggested for this pathology. In fact, similar to AD, stimulating caspase-1 activation and the release of IL-1β is necessary to induce the production of ROS and activity of cathepsin-B^110^. Accordingly, through specific inhibition of cathepsin-B, it is possible to interfere with the inflammasome assembly. However, an *in vivo* animal model did not validate the findings of this study and experiments were only performed in monocytes. More relevant to this study, Yan et al. showed that dopamine-producing neurons and the NLRP3 inflammasome are tightly interconnected and are able to regulate each other^111^. Intriguingly, the authors demonstrated that the neurotransmitter dopamine (DA) has the potential to inhibit NLRP3 inflammasome activation and subsequent IL-1β production and that the mechanism underlying the inhibitory activity of DA on NLRP3 occurs via the dopamine D1 receptor (DRD1) signaling through an autophagic-dependent process. Most importantly, experiments in transgenic mice deleted for DRD1 or NLRP3 and treated with MPTP showed that DRD1 signaling counteracts MPTP-induced neuroinflammation by inhibiting the NLRP3 inflammasome. In addition, authors also found an important role for DA and DRD1 in regulating lipopolysaccharide-induced systemic inflammation and MSU-induced peritoneal inflammation^111^. Overall, these data highlight the possibility to consider DA and DRD1 as potential targets for counteracting inflammation in AD.

Insulin resistance and islet β-cell dysfunction in type 2 diabetes (T2D) are widely associated with disruptions of MAM compartments (Fig. 4). The group coordinated by J. Rieusset clearly demonstrated a strong relationship between MAM integrity and efficient insulin action in hepatic cells, indicating MAMs as novel actors in the mechanism of action of insulin liver^112,113^. Indeed, they demonstrated in vitro and in vivo that defective ER–mitochondria coupling is closely associated with impaired hepatic insulin sensitivity and restoration of MAM integrity by cyclophilin D overexpression improves insulin signaling in primary hepatocytes of diabetic mice^113^.

Accordingly, in the skeletal muscle of obese and diabetic humans, the expression levels of the ER–mitochondria tethering protein MFN2 are reduced. At confirmation, in vivo experiments demonstrated that feeding animals with high-glucose diet reduced MFN2 expression and attenuated insulin signaling that is reduced by promoting MFN2 overexpression. These processes appeared to be driven by a significant downregulation of mitochondrial activities. Indeed, the livers of transgenic mice deleted for MFN2 possessed a low insulin response and high number of fragmented mitochondria that reflected a reduction in mitochondrial respiration due to an atypical functioning of OXPHOS complex subunits. As a consequence, ROS production increased with subsequent accumulation of mutation at the level of mtDNA^114^. Increases in ROS levels appeared to be a primary contributor to inflammation in T2D. In fact, pro-inflammatory cytokines improved ER and oxidative stress events, leading to β-cell loss, recruitment of NLRP3 inflammasome, and finally, to the pathogenesis of T2D. Different molecular pathways have been proposed to work on this process. As previously described, increased ROS production triggers conformational changes in TXNIP and subsequent loss of the complex TRX–TRXNIP that binds and activates NLRP3, which produces IL-1β secretion^31^.

Interestingly, a recent study in human monocytes demonstrated that high glucose causes excess ROS production and TXNIP-mediated NLRP3 inflammasome activation through TRPM2-mediated Ca^2+^ influx and p47 phox signaling^115^; as such, TXNIP1 could be considered a potential therapeutic target for diabetes^94^.

Alternatively, a recent study suggested that ROS might activate NLRP3 via the action of intracellular AMP-activated protein kinase (AMPK) in an autophagic-dependent manner^116^. Notably, AMPK is a crucial mediator of metabolism of fatty acids and inhibits ROS production by regulating the expression and function of NADPH oxidase. In this elegant study, the authors demonstrated that a diet rich in saturated fatty acids promotes the inhibition of AMPK activation and reduction in autophagic activity. When the autophagic machinery is silenced, the ROS production increases and activates the NLRP3 inflammasome^116^.

MAMs and mitochondrial dynamics are also recognized as key factors in the pathogenesis of cardiac and vascular diseases (Fig. 4). The first support for this concept was found in the early 1990s, when it was demonstrated that the precise Ca^2+^ transport from the ER to the mitochondria regulates the cardiac contraction cycle^117^. Subsequent studies demonstrated that mitochondrial Ca^2+^ fluctuations and Ca^2+^ oscillation triggered by ER are present during cardiomyocyte beating^118^. Later, it was demonstrated that the precise transport from the ER to mitochondria is widely regulated by the appropriate composition of the MAMs fraction^118^. Among the proteins involved in the maintenance of MAMs, MFN1/2 seem to be the most relevant ones^119^. In fact, adult hearts deleted for both mitofusins showed compromised cardiac function, augmented left ventricular end-diastolic volume, and reduced fractional shortening^120^. In addition, the transgenic *MFN2*^−/−^ mice exhibited reduced contact length between these organelles, a reduction in the ER-mitochondrial Ca^2+^ transfer, and increased ROS production.

Although only few studies identified a specific role for MAMs in cardiovascular diseases (CVD), it seems that these reactive chemical species are important contributors for different CVD. Interestingly, excessive ROS production and subsequent NLRP3 activation are frequently found in CVD, such as atherosclerosis (AS), where an excess of cholesterol is deposited in the arterial wall as cholesterol crystals leading to inflammatory injury. It has been demonstrated that, by internalizing these crystals, macrophages promote NLRP3 inflammasome activation in a process involving leakage of cathepsin B and L into the cytoplasm, excessive formation of mitochondrial ROS, and lowering in potassium concentrations^121^.

The important role of inflammasomes was confirmed in AS using *ApoE*^*−/−*^ mice (transgenic mouse model of AS, which develop spontaneous AS when fed with a high-fat diet); deletion of IL-1β reduced the size of atherosclerotic lesions by up to 30%^122^. Moreover, the deletion of the IL-18 receptor (*IL-18R*^*−/−*^) decreased the size of the lesions^123,124^. Despite this, NLRP3 may be not the only source of pro-inflammatory cytokines in AS. In fact, transgenic mice ApoE^*−/−*^ crossed with mice deleted for different components of the NLRP3 (*Nlrp3*^*−/−*^, *Asc*^*−/*^^−^, or *caspase-1*^*−/−*^) exhibited no differences in atherosclerotic lesions and plaques when compared to the double-knockout and control mice^125^. As such, further studies to elucidate the implication of NLRP3 inflammasome in AS are needed.

NLRP3 inflammasome recruitment and the appropriate MAM composition also have an important role during ischemia/reperfusion (I/R). Notably, IL-1β and IL-18 are primary mediators of I/R-induced human myocardial injury, and through the inhibition of caspase-1 activity it is possible to reduce the depression in contractile force after I/R in an I/R model^126^. Similarly, in *ASC*^*−/−*^ mice the amount of inflammatory cytokines and resulting injuries such as the development of infarctions, myocardial fibrosis, and dysfunction in myocardial I/R injury were significantly reduced compared to wild-type controls^127^. Finally, it has been reported that specific proteins conserving the ER–mitochondria interface are involved in I/R. For example, OPA1 deficiency was associated with increased sensitivity to I/R, whereas the inhibition of FIS1 and DRP1 function was reported to be cardioprotective^128^.

To summarize, it is necessary to improve the understanding of the role that MAMs and inflammasome activation play in CVD. Nevertheless, the data suggest that both mechanisms may be considered potential therapeutic targets. Accordingly, treatments aimed to modulate ER–mitochondrial dynamics have been developed to target or treat cardiovascular diseases, and specific drugs counteracting the activity of components of inflammasome are available.

## Conclusions

Accumulating evidence indicates that ER–mitochondria contact sites play important roles in promoting inflammation and the development of inflammatory diseases.

However, the new understanding of how MAMs could impact inflammatory signaling raises several questions. Can MAMs be attractive targets for bacterial proteins? Several important processes regulated at MAM level are modulated by pathogens to subvert host functions and promote infection, thus it is tempting to assume that pathogenic bacteria target MAMs to undermine key host cellular processes.

Importantly, inflammasome activity is crucial for the host response to microbial pathogens and possibly for optimal response to vaccine adjuvants.

Recent studies have shed light on MAM dynamics as critical regulators/effectors of antiviral signaling^129^; however, the function of MAM-mediated antiviral signaling deserves further investigation. With the current knowledge, MAMs appear to be a recruitment platform involved in the antiviral pathway. However, are other MAMs proteins, such as MAVS, required for this signaling for a proper antipathogen response and/or inflammatory response? Do the MAMs also transduce other innate immunity signaling? Future studies should provide answers to some of these interesting questions.

With regard to inflammasome activity, NLRP3 and other inflammasome members locate to ER–mitochondria sites to promptly sense the level of damage and to coordinate the appropriate response. In fact, not all inflammasome activation can be considered harmful, and the therapeutic inhibition of this pathway must be balanced with its beneficial contribution.

Importantly, a deeper understanding of the balance between beneficial and damaging inflammasome activation is also required in order to create new therapies for patients with inflammatory diseases.

Hence, increasing our knowledge regarding the molecular aspects and functions of different inflammatory players at MAMs is the only way to provide future therapeutic targets for a wide range of inflammatory diseases and to ameliorate patient outcomes.
